# Publications in BJS and BJS Open by Professor Henrik Kehlet, inaugural winner of the BJS Society Award in Surgery

**DOI:** 10.1093/bjsopen/zrad078

**Published:** 2023-09-05

**Authors:** Jonothan J Earnshaw

**Affiliations:** Director of BJS Academy and former Editor-in-Chief of BJS


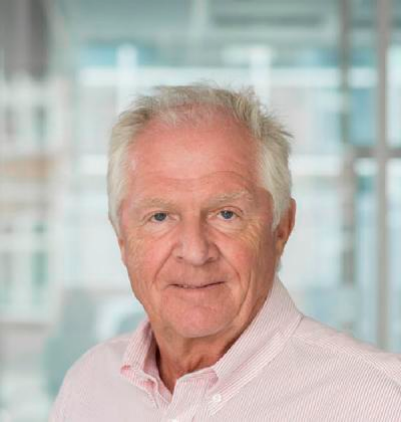


**Professor Henrik Kehlet**

Although not specifically part of the assessment process, the fact that Professor Henrik Kehlet has co-authored (so far) 46 peer-reviewed or invited articles published in BJS, during his distinguished career, makes him a worthy winner of the inaugural BJS Society Award.

His publications in BJS span more than 35 years. His first paper, published in 1978, was typical of his scientific approach and examined the physiological stress induced by cholecystectomy and suggested intervention with saline could reduce the aldosterone response^1^. His penultimate paper, published in 2013, evaluated a multimodal postoperative care programme for patients undergoing liver resection and showed discharge could be achieved a median of 2 days after laparoscopic resection and 4 to 5 days after open resection^2^. This was the culmination of three decades of research looking at the many aspects of improving perioperative care, which has currently become a multimodal care package, known as enhanced recovery.

Professor Kehlet’s last BJS paper, published in 2015, used information from the Danish Hernia Database that he helped to start in 1997^3^. It showed that the method of mesh fixation during incisional hernia repair mattered, with absorbable tacks inferior to non-absorbable tacks. After it was founded, he was responsible for a number of papers using the Danish Hernia Database to study existing methodology and the move towards minimally invasive hernia surgery.

Professor Kehlet’s most cited paper in BJS was also the one he refers to as his favourite in his recent interview. The paper, published in 2000, suggested paralytic ileus was preventable^4^. It is still worth a read more than two decades on. In a detailed review, he and his co-authors explained postoperative systemic changes in detail and how they might be prevented. It was received with some scepticism, particularly in North America, but has since been cited 498 times and multimodal enhanced recovery has become the standard of care.

Two of his other papers were each cited over 400 times. The first, published in 1999, was a proof-of-concept paper concerning 16 patients, showing that it was possible to discharge them 2 days after open sigmoid colectomy (459 cites)^5^. The second (440 cites), published in 2007, concluded that having an agreed protocol was not enough to ensure optimal perioperative care^6^. The paper concluded that, to be effective, enhanced recovery needs structured organizational change for the benefits to be realized. This has evolved into the modern perioperative care pathway.

One other paper deserves a mention, as it has been viewed more than 1200 times. The paper on evidence-based management of postoperative pain after open inguinal hernia repair was a review of 334 RCTs and emphasized the importance of instilling local anaesthetic into the surgical wound and using postoperative analgesia with anti-inflammatory drugs combined with paracetamol^7^. Although it was only cited 101 times it too is the accepted standard of care to this day.

Professor Kehlet has also published three papers in BJS Open, the open access companion journal to BJS. In 2022 he published two papers on video assisted thoracotomy, showing that his work is moving with the times towards less invasive surgery.

Professor Kehlet has had a long and varied publishing career, with over 1200 co-authored papers on PubMed. His themed research into hernias was important during the evolution of minimally invasive intervention. Perhaps, even above this, he can be considered a giant in the field of enhanced recovery after surgery.

Few surgeons have published more than 46 papers in BJS and this alone is a reason to congratulate him on his BJS Society Award in 2023. Click here for the full list of Professor Kehlet’s publications in BJS. Click here to access his BJS Open publications.

## Notes

*Since this article was written, Professor Kehlet has published his 50^th^contribution in BJS/BJS Open, responding to current concerns over the safety of same-day discharge after surgical procedures. Long may he continue to contribute his wisdom and experience to this topic.*
